# Effects of Emulsifiers on Physicochemical Properties and Carotenoids Bioaccessibility of Sea Buckthorn Juice

**DOI:** 10.3390/foods13131972

**Published:** 2024-06-22

**Authors:** Arzigül Abliz, Yanan Huang, Reziwanguli Rouzi, Duoxia Xu, Yanxiang Gao, Jinfang Liu

**Affiliations:** 1 Key Laboratory of Healthy Beverages, China National Light Industry, College of Food Science & Nutritional Engineering, China Agricultural University, Beijing 100083, China; arzigulabliz@cau.edu.cn (A.A.); 2019309080122@cau.edu.cn (Y.H.); reziwanguli@cau.edu.cn (R.R.); gyxcau@126.com (Y.G.); 2 Beijing Engineering and Technology Research Center of Food Additives, Beijing Technology and Business University, Beijing 100048, China; xuduoxia@th.btbu.edu.cn

**Keywords:** sea buckthorn juice, dynamic high pressure microfluidization, emulsifier, carotenoids, bioaccessibility

## Abstract

The need to improve the physicochemical properties of sea buckthorn juice and the bioavailability of carotenoids is a major challenge for the field. The effects of different natural emulsifiers, such as medium-chain triglycerides (MCTs), tea saponins (TSs) and rhamnolipids (Rha), on the physical and chemical indexes of sea buckthorn juice were studied. The particle size of sea buckthorn juice and the carotenoids content were used as indicators for evaluation. The effects of different addition levels of MCT, Rha and TS on the bioavailability of carotenoids in sea buckthorn juice were investigated by simulating human in vitro digestion tests. The results showed that those emulsifiers, MCT, Rha and TS, can significantly reduce the particle size and particle size distribution of sea buckthorn juice, improve the color, increase the soluble solids content, turbidity and physical stability and protect the carotenoids from degradation. When the addition amount of Rha was 1.5%, the total carotenoids content (TCC) of sea buckthorn juice increased by 45.20%; when the addition amount of TS was 1.5%, the total carotenoids content (TCC) of sea buckthorn juice increased by 37.95%. Furthermore, the bioaccessibility of carotenoids was increased from 36.90 ± 2.57% to 54.23 ± 4.17% and 61.51 ± 4.65% through in vitro digestion by Rha and TS addition, respectively. However, the total carotenoids content (TCC) of sea buckthorn juice and bioaccessibility were not significantly different with the addition of MCT. In conclusion, the findings of this study demonstrate the potential of natural emulsifiers, such as MCT, Rha and TS, to significantly enhance the physicochemical properties and bioavailability of carotenoids in sea buckthorn juice, offering promising opportunities for the development of functional beverages with improved nutritional benefits.

## 1. Introduction

Sea buckthorn (*Hippophae rhamnoides* L.) is a kind of super fruit rich in nutrients and biologically active ingredients such as carotenoids, phenols, flavonoids, vitamins and minerals [1,2,3]. In sea buckthorn, carotenoids are not only the main components that contribute to the color of sea buckthorn but are also health-promoting compounds [4,5]. Carotenoids contribute to prevent or ameliorate chronic diseases in the body, such as cancer, cardiovascular disease and skin and eye diseases [6,7,8]. However, sea buckthorn juice has a mixed and unstable system due to its fatty acids content and fine pulp particles, resulting in unfavorable phase separation in the product. Furthermore, carotenoids as the valuable nutrients and colorants in sea buckthorn juice are sensitive; therefore, degradation is prone to occur during the processing of sea buckthorn juice [9,10].

Dynamic high-pressure microfluidization (DHPM) is a new high-pressure homogenization technology that is used in the field of food emulsification homogenization, macromolecular modification, extraction of active substances, preparation of liposomes and nanoemulsions, wet superfine grinding and sterilization [11,12,13]. DHPM uses microchannels to form a high-speed jet, making the fluid suffer high-speed shock, strong shear, high-frequency vibration, instantaneous pressure drops, and hydrodynamic cavitation within a short treatment time, thus providing a much higher energy density compared with conventional valve homogenization [14,15,16]. In fruit and vegetable juice/pulp processing, the application of DHPM technology is beneficial for obtaining natural fruit or vegetable juice/pulp with enhanced nutritional properties [17,18]. For example, DHPM treatment can reduce the particle size and change the properties of tomato solids in tomato paste and increase the content of detectable lycopene in tomato paste [19].

Food emulsifiers are a kind of high-efficiency and multifunctional food additive that play a crucial role in the physical and chemical properties of natural and processed foods, with the functions of emulsification, stabilization, dispersion, thickening, shortening, lubrication, defoaming or foaming and protection [20]. Food emulsifiers decrease the surface tension between the various constituent phases in the mixture liquid to form a uniform emulsification system, thereby improving the structure of the food, stabilizing the physical state of the food, improving the flavor and mouthfeel of the food and improving the quality of the food [21,22]. Choosing appropriate food emulsifiers or stabilizers in fruit and vegetable juice can help to solve the problem of fat rising or stratification caused by the insolubility of some components in plant raw materials or the fruit and vegetable juice matrix, form a stable homogeneous solution and improve sensory quality. Medium-chain triglycerides (MCTs), tea saponins (TSs) and rhamnolipids (Rha) are emulsifiers of natural origin with good stability and emulsifying properties that are also healthy and environmentally friendly. By studying their effects on sea buckthorn juice, their potential applications in the development of functional beverages can be evaluated.

It has been reported that homogenization can delay the phase separation of juice [9]. High-pressure homogenization (HPH) has been used to improve the physical stability of emulsions and other plant-based products [23]. During homogenization, the material is subjected to mechanical forces, such as shear forces or local pressure fluctuations, leading to the disruption of cells and droplets [24]. This leads to a reduction in particle size, which will increase the suspension of the particles and increase the stability of the product. Based on previous research from our team, dynamic high-pressure microfluidization (DHPM) has significant advantages in improving physical stability and the carotenoid release of sea buckthorn juice compared to conventional homogenization [25]. To avoid the potential health risks of artificially synthesized emulsifiers, natural emulsifiers are more popular among consumers in recent days. Therefore, there is an urgent need to investigate the potential benefits of natural emulsifiers in combination with DHPM to optimize the nutritional content and bioavailability of carotenoids in sea buckthorn juice while ensuring product safety.

The purpose of this study is to improve the nutritional quality of sea buckthorn juice, improve the bioaccessibility of carotenoids and meet the health needs of consumers by combining dynamic high-pressure microfluidization (DHPM) with emulsifiers while ensuring product safety. The effects of different natural emulsifiers, such as medium-chain triglycerides (MCTs), tea saponins (TSs) and rhamnolipids (Rha), on the physical and chemical indexes of sea buckthorn juice were studied. The particle size of sea buckthorn juice and the carotenoids content were examined and compared. The juices were stored at −18 °C.

## 2. Materials and Methods

### 2.1. Materials and Chemicals

The sea buckthorn turbid juice (raw) was supplied by Beijing Powdery Health Industry Co., Ltd. (Beijing, China). MCT was provided by Musim Mas Co., Ltd. (Singapore). Rhamnolipids were provided by Shaanxi Panier Biotechnology Co., Ltd. (Xi’an, China). Tea saponin was provided by Xi’an Zelang Biological Technology Co., Ltd. (Xi’an, China). Other chemical reagents were purchased from Beijing Chemical Plant Co., Ltd. (Beijing, China) or Sinopharm Chemical Reagent Co., Ltd. (Shanghai, China).

### 2.2. DHPM Treatment of Sea Buckthorn Juice

The raw sea buckthorn juice was filtered through a 100-mesh nylon filter cloth, labeled as ND. Then, different emulsifiers (MCT, Rha, TS) were added into the filtered sea buckthorn juice with different proportions (0%, 0.05%, 0.10%, 0.15% and 0.20% for MCT, 0%, 0.5%, 1.0%, 1.5%, 2.0% and 2.5% for Rha, 0%, 0.1%, 0.2%, 0.3%, 0.4%, 0.5% and 1.0%, 1.5%, 2.0%, 2.5% for TS). The addition proportions and types of emulsifiers are also shown in Table S1. The juice added with MCT, after preheating in a water bath at 50 °C for 10 min, was homogenized at 8000 rpm for 2 min (Ultra Turrax, model T25, IKA Labortechnik, Staufen, Germany) to ensure adequate mixing. For the samples added with Rha and TS, the emulsifiers were dissolved using a magnetic stirrer for 10 min until completely dissolved in the raw sea buckthorn juice. Except for control (labeled as ND), all the samples were subjected to homogenization under 150 MPa and one pass using a Y-type reactor (Microfluidizer^®^ processor model M-110EH, Microfluidics Inc., Newton, MA, USA), and the treated samples were labeled in the form of concentrations with emulsifier type (0% refers to treated with DHPM without emulsifier) (shown as Table S1).

### 2.3. Particle Size Measurement

The particle size distribution (PSD) of sea buckthorn juice was evaluated by LS 230 laser particle size analyzer (American Beckman Coulter Co., Ltd., Miami, FL, USA). The optical properties were evaluated as follows: a refractive index of 1.362 for sea buckthorn juice according to refractive index measurement using refractometer and absorption of 0.01, and a refractive index of 1.333 for the dispersant.

### 2.4. ζ-Potential Measurement

All the samples were diluted 20 times with distilled water, and then the ζ-potential was measured at room temperature by Zetasizer Nano-Zs 90 laser particle size analyzer (Malvern Instruments, Worcestershire, UK).

### 2.5. Turbidity, Total Soluble Solids Content and pH Measurement

All samples were diluted 20 times with a citric acid–sodium citrate buffer solution (a buffer solution of the corresponding pH value was prepared based on the pH of the sample). The turbidity of the samples was measured at room temperature using a 2100 N turbidimeter (HACH Water Analysis Instrument Co., Ltd., Loveland, CO, USA), and the turbidity unit was expressed as NTU [26].

Total soluble solids (TSS) was determined using an Abbemat 500 digital refractometer (Anton Paar Trading Co., Ltd., Shanghai, China) at 20 ± 1 °C, and results were expressed as °Brix.

The pH values were measured by using a portable pH meter (Orion 3Star, Thermo Fisher Scientific Co., Ltd., Waltham, MA, USA) at 25 ± 1 °C.

### 2.6. Physical Stability

The physical stability of sea buckthorn juices was determined using a LUMiSizer (L.U.M. 290 GmbH, Berlin, Germany) based on the established principle that uses centrifugal sedimentation to accelerate the occurrence of instability phenomena such as sedimentation, flocculation and creaming [27]. The measurements were carried out under the following conditions: the rotation speed was 4000 rpm, the acquisition time was 5400 s at 25 °C and the time intervals were 20 s.

### 2.7. Observation of Microstructure

A total of 20 µL of sample (sea buckthorn fruit removed from peels, raw juice and the treated juice samples) was placed onto a clean glass slide, and then the cover slide was covered and observed immediately. Images were obtained under eyepiece ×10 and objective ×100 using Leica optical microscope (LEICA DM500, Leica Instrument Co., Ltd., Berlin, Germany).

### 2.8. Color

A small amount of sample was placed in an appropriate container, and the color parameters, L*, a*, b* and ΔE, were measured using colorimeter (high-quality colorimeter NH300, 3NH technology Co., Ltd., Shenzhen, China) [28,29].

### 2.9. Determination of Carotenoids Content

The extraction of carotenoids was based on the methods of previous article with some modifications [30]. Seven-gram samples were weighed accurately (to the nearest 0.001 g) and then mixed with 8.4 mL extract solutions (dichloromethane: methanol: acetone = 2:1:1, 0.1% BHT). The sample was thoroughly mixed with the carotenoid extract and centrifuged at 10,000 rpm for 30 min at 4 °C. The extraction step was repeated twice and the two parts of the organic phases were combined and then washed with distilled water. Carotenoids were extracted after concentration and drying of the organic phase using a nitrogen thickener (MTN-2800W Nitrogen blowing thickener, Tianjin Automatic Science Instrument Co., Ltd., Tianjin, China). The extraction of carotenoids was redissolved in petroleum ether and the absorbance was measured at 450 nm using a spectrophotometer (UV-1800-type UV–visible spectrophotometer, Shimadzu, Kyoto, Japan). The total content of carotenoids was calculated using a standard curve. The carotenoid content of sea buckthorn juice without the addition of emulsifiers is blank.

### 2.10. In Vitro Digestion

The release rate and bioaccessibility of carotenoids from sea buckthorn juice were determined through an INFOGEST statical ternary-phase in vitro digestion experiment (including mouth, stomach and small intestine digestion) with reference to the anterior research [31,32].

In brief, 25.0 g (volume of 22.5 mL) of sea buckthorn juice was mixed with 25.0 g (volume of 24.5 mL) of oral digestive juice (SSF) in a 1:1 (*w*/*w*) ratio. The mixture was placed in an incubator at 37 °C for 2 h and shaken at 100 rpm. In the gastric digestion phase (pH 3.0), 40 mL of the oral digestive fluid was mixed with 32 mL of the gastric digestive juice (SGF). The enzyme activity of the added pepsin in the final mixed system was 2000 U/mL and was digested at 37 °C for 2 h with shaking at 100 rpm. Subsequently, 70 mL of gastric digesta was mixed with 54 mL of small intestinal digestive juice (SIF) (pH 7.0), and pancreatin (trypsin activity 100 U/mL) and bile salt (54 mg/mL) were added and digested at 37 °C for 2 h with shaking at 100 rpm.

The digesta was collected every 30 min during the whole digestion procedure for further analysis. A small amount of samples were taken from each digestion stage and stained, and the microstructure was observed using a laser confocal scanning microscope (CLSM). According to the method in Section 2.9, carotenoid content was determined using UV-1800-type UV–visible spectrophotometer.

The bioaccessibility of carotenoids in sea buckthorn juice was assessed after the whole simulated digestion procedure. The supernatant of digesta obtained by centrifugation of the small intestinal digest at 11,000 r/min and 4 °C for 60 min was considered as having a mixed micelle phase. The supernatant was also subjected to filtration (the pore size of the filter membrane was 0.22 μm) before the concentration determination. The concentration of carotenoids solubilized in the micellar phase was determined spectroscopically according to the method described in Section 2.9, and the bioaccessibility (%) of carotenoids in sea buckthorn juice was estimated by following the equation below:

(1) $$\mathrm{Bioaccessibility}\;(\%)=\frac{C_{micelle}}{C_{initial\;rehydrated\;suspension}}\times 100\%$$

where C*_micelle_* and C*_initial_* suspension represent the content of carotenoids in mixed micellar phase and the total content of carotenoids in the initial rehydrated suspension, respectively.

### 2.11. Statistical Analysis

The results were expressed as mean ± SD and then subjected to statistical analysis of variance using SPSS 22.0 for Windows (SPSS Inc., Chicago, IL, USA). Statistical differences were determined by one-way analysis of variance (ANOVA) with Duncan’s post hoc test, and least significant differences (*p* < 0.05) were accepted among the treatments.

## 3. Results and Discussion

### 3.1. The Particle Size Distribution of Sea Buckthorn Juice

**Figure 1:**
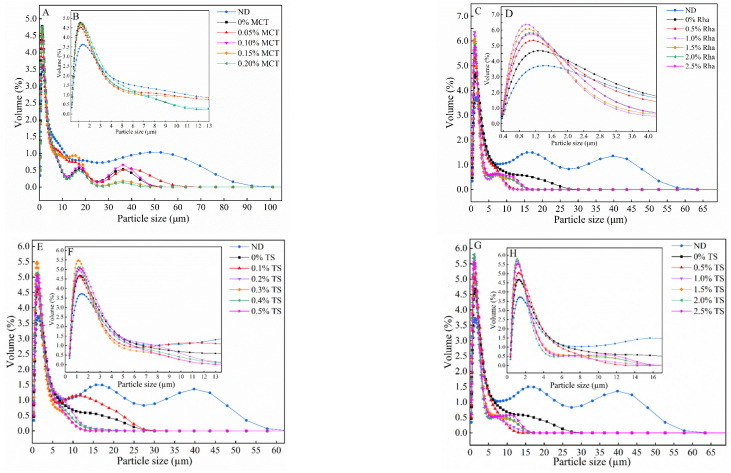
Effect of combined treatment with DHPM and emulsifier on the PSD of sea buckthorn juice: (**A**,**B**), overall and partially magnified view of the PSD of sea buckthorn juice added with MCT, respectively; (**C**,**D**), overall and partially magnified view of the PSD of the samples added with Rha, respectively; (**E**,**F**), overall and partially magnified view of the PSD of the samples added with low-concentration TS, respectively; (**G**,**H**), overall and partially magnified view of the PSD of the samples added with high-concentration TS, respectively.

The influence of MCT, Rha and TS in different concentrations on the PSD of sea buckthorn juice is shown in Figure 1. Figure 1A,B show that the PSD of sea buckthorn juice to which MCT was added is distributed in a multi-peak pattern consisting of a major peak and two minor peaks. The size of the major peak is in the range of 0.4~12.0 μm, and the corresponding size of the minor peak is about 1.3 μm. When the MCT concentration is 0~0.20%, the main peak shifts to the left and the particle size decreases compared to ND. However, the major peaks of 0%MCT ~0.20%MCT almost overlap (see Figure 1B), and the minor peaks are slightly different.

For the PSD of sea buckthorn juice to which Rha was added, there were three peaks in the PSD of ND, with particle size distributions of 0.4~7.5 μm, 7.5~27.0 μm and 27.5~63.0 μm, respectively (see Figure 1C). The PSDs of 0% Rha and 0.5% Rha were unimodal peaks, and the PSDs of 1.0% Rha to 2.5% Rha were bimodal peaks. At 0% Rha~1.0% Rha, the particle size corresponding to the peak of the main peak decreased, the volume fraction increased (the volume fraction was 3.70%, 4.70%, 5.38% and 6.42%, respectively, separately) and the peak of the particle size distribution gradually narrowed (see Figure 1D). At 1.0% Rha~2.5% Rha, the size corresponding to the peak increased and the volume fraction decreased (6.42%, 6.08%, 5.83% and 5.76%, respectively, separately).

In the PSD of sea buckthorn juice to which a low TS concentration (0~0.5%) was added, the volume fraction corresponding to the peak of the main peak increased from 0% TS to 0.3% TS and decreased from 0.3% TS to 0.5% TS compared to ND (see Figure 1E,F). The result was the same as that of Rha, i.e., the particle size of sea buckthorn juice spiked with a low TS concentration, initially decreasing and then gradually increasing. Since the particle size of the major peak is smaller than that of the minor peak, it can be concluded that when 0~0.3% TS is added, the smaller particles will increase. The PSD of sea buckthorn juice to which a higher TS concentration (0.5~2.5%) was added shifted toward the smaller particle size compared to 0% TS, the peak volume fraction corresponding to the major peak increased with the increase in TS concentration and the major peak overlapped when the addition amount is 2.0% and 2.5% (see Figure 1G,H).

The mean particle size of the samples also changed with the DHPM treatment and the different emulsifiers. The changes in mean particle size show the same trend as the PSD (see Supplementary Material). The particle size of the colloidal system is closely related to the stability of the system [14]. The most common stability problems in plant-based fruit and vegetable juices are the emulsion stratification of oil droplets and the sedimentation of solid particles. Since the settling rate of small particles is slower than that of large particles, a system composed predominantly of small particles may be more stable than a system composed predominantly of relatively large particles. In this study, the sedimentation of the particles could be related to the effect of emulsifier addition on the recombination of the surface structure. In addition, the Rha and TS used in this study have relatively strong hydrophilic properties and the MCT has relatively strong oleophilic properties [33]. Hydrophilic emulsifiers dissolve easily in the water phase and sea buckthorn juice can be considered as having a water phase system. When the amount of emulsifier in the aqueous system is small, one molecule of emulsifier is close to more than one molecule of oil and water in order to form oil granules. When the amount of emulsifier is high, several small emulsifier molecules are in the vicinity of one oil molecule and form milk granules [34]. Since the particle size of oil particles is larger than that of milk particles, the particle size of the system decreases with a rise in emulsifier concentration and drop in surface load [35,36].

### 3.2. ζ-Potential

**Figure 2:**
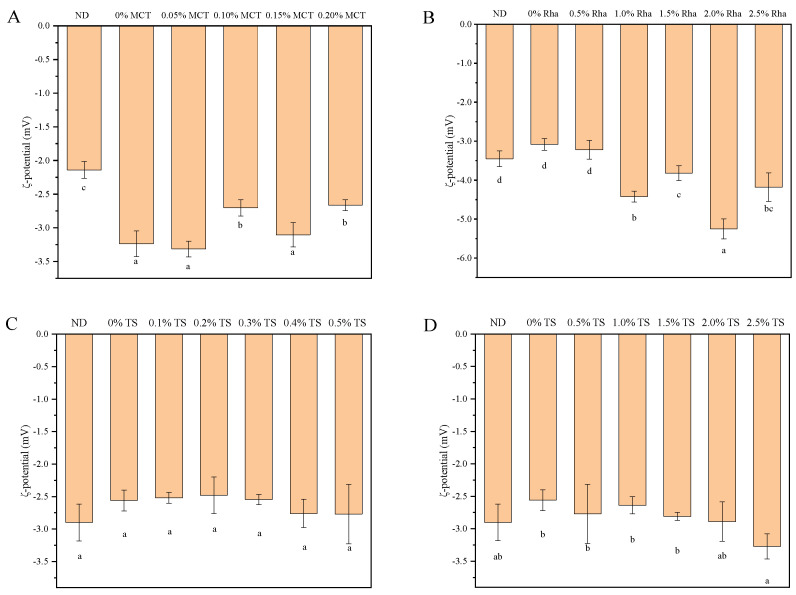
The effect of different emulsifiers on the ζ-potential of sea buckthorn juice: (**A**), MCT; (**B**), Rha; (**C**,**D**), low-concentration TS and high-concentration TS, respectively. Notes: lower-case labels indicate the significance of differences between samples (*p* < 0.05).

The ζ-potential is an important indicator for evaluating emulsion stability. Higher ζ-potential values indicate that the system is stable since electrostatic repulsion prevents liquid droplet aggregation [37]. The influence of different emulsifiers on the ζ-potential of sea buckthorn juice is shown in Figure 2. Compared with 0% MCT, the value of the ζ-potential of 0.20% MCT and 0.10% MCT decreased significantly, and the value of the ζ-potential of 0.05% MCT and 0.10% MCT did not change significantly (Figure 2A). For the sea buckthorn juice added with Rha, the absolute value of ζ-potential increased significantly when the addition amount of Rha is greater than 1.0%, and the absolute value of the ζ-potential of 2.0% Rha was the largest (Figure 2B). The differences between the emulsion ζ-potential values were negligible in a range of the amount of TS was 0.1~2.5%, indicating that TS changes in a narrow range did not have a significant impact (Figure 2C,D). The value of the ζ-potential of 2.5% TS was significantly increased compared with 0% TS, which is consistent with the results of previous research [38]. ζ-potential is related to the charge of the sample. Hydrophilic emulsifiers contain more –OH groups and exhibit a higher potential intensity upon binding. Moreover, the higher potential charge provides a stronger repulsive effect to maintain droplet independence [39]. Therefore, the reason for the insignificant change in ζ-potential in sea buckthorn juice with MCT and TS may be related to the types of emulsifiers; that is, MCT and TS are non-ionic emulsifiers and Rha is an anionic emulsifier [40,41].

### 3.3. Turbidity, pH and Total Soluble Solids Content

**Figure 3:**
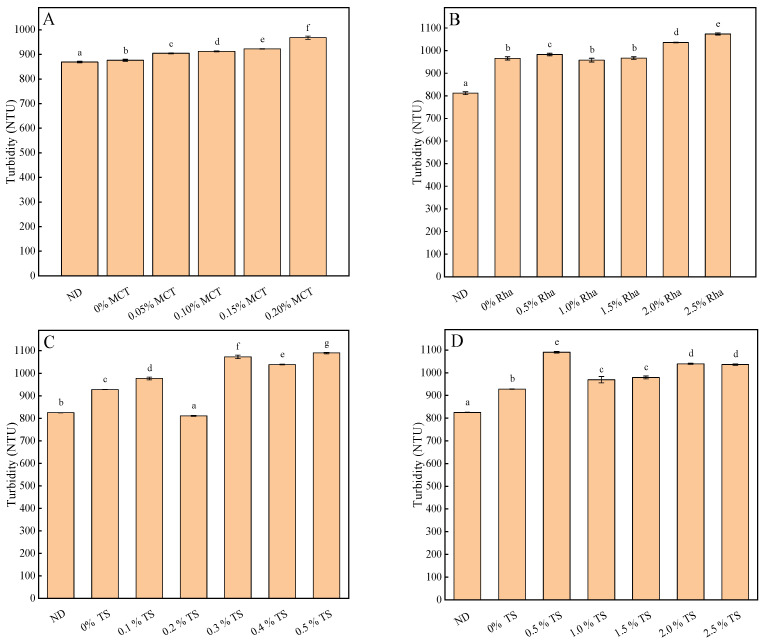
The influence of different emulsifiers on the turbidity of sea buckthorn juice: (**A**), MCT; (**B**), Rha; (**C**,**D**), low-concentration TS and high-concentration TS, respectively. Notes: lower-case labels indicate the significance of differences between samples (*p* < 0.05).

The influence of different emulsifiers on the turbidity of sea buckthorn juice is shown in Figure 3. Turbidity increased significantly with the increase in MCT content shown in Figure 3A. For the sea buckthorn juice added with Rha, turbidity varied with the amount of Rha, and the results are shown in Figure 3B. The turbidity increased significantly when the amount of Rha was greater than 1.5% compared with 0% Rha. When a low concentration and high concentration of TS were added into sea buckthorn juice, the turbidity varied with different TS concentrations (shown as Figure 3C,D), which was similar to the result of Rha. The difference in turbidity gradually decreased as the concentration of each emulsifier increased. These phenomena may be caused by the different particle compositions at the interface of emulsions prepared with different emulsifiers and by the slightly different colors of nanoemulsions prepared with different emulsifiers [42]. In brief, turbidity is related to emulsifier type and concentration [43]. These results indicate that the amount of emulsifier is closely related to the recombination of the interface in the system. Also, the data indicate that smaller particle sizes achieved through the addition of emulsifiers correlate with increased turbidity. For example, while MCT and Rha reduced particle size significantly, turbidity increased, suggesting denser droplet packing, which enhances light scattering, affecting the juice’s visual clarity and consumer appeal. Notably, further reductions in particle size with higher concentrations of Rha did not proportionally increase turbidity, indicating a potential saturation of the emulsifier’s effect. With TS, the trend of increasing turbidity at higher concentrations aligns with the smallest particle sizes observed, reinforcing the link between finer emulsions and increased turbidity. This suggests that while smaller droplet sizes improve emulsion stability, they can also lead to higher turbidity, which might compromise the aesthetic appeal of the juice. This analysis highlights the need for a balanced approach in emulsifier concentration to enhance stability while maintaining acceptable turbidity levels, crucial for consumer acceptance and the commercial success of functional beverages like sea buckthorn juice.

**Figure 4:**
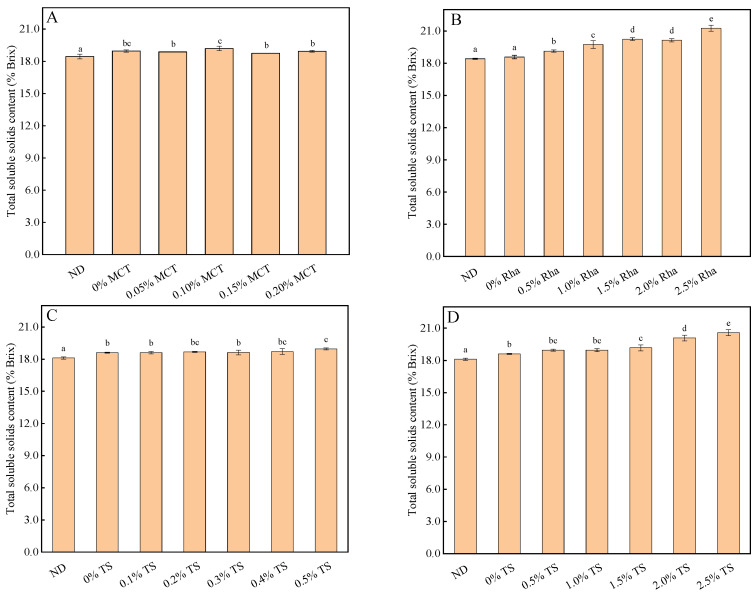
The influence of different emulsifiers on the effect of the TSS of the sea buckthorn juice: (**A**), MCT; (**B**), Rha; (**C**,**D**), low-concentration TS and high-concentration TS, respectively. Notes: lower-case labels indicate the significance of differences between samples (*p* < 0.05).

The effects of different emulsifiers on the TSS of sea buckthorn juice are shown in Figure 4. Except for 0.10% MCT, the TSS of 0.05% MCT, 0.15% MCT and 0.20% MCT showed no significant change compared with 0% MCT (shown in Figure 4A). TSS increased significantly with the increase in Rha content when 0~2.5% Rha was added in sea buckthorn juice (shown in Figure 4B). The TSS in sea buckthorn juice changed little when 0~1.0% TS was added (shown in Figure 4C). However, when the concentration of TS was increased to 1.5~2.5%, TSS was significantly increased compared with 0% TS (shown in Figure 4D).

**Figure 5:**
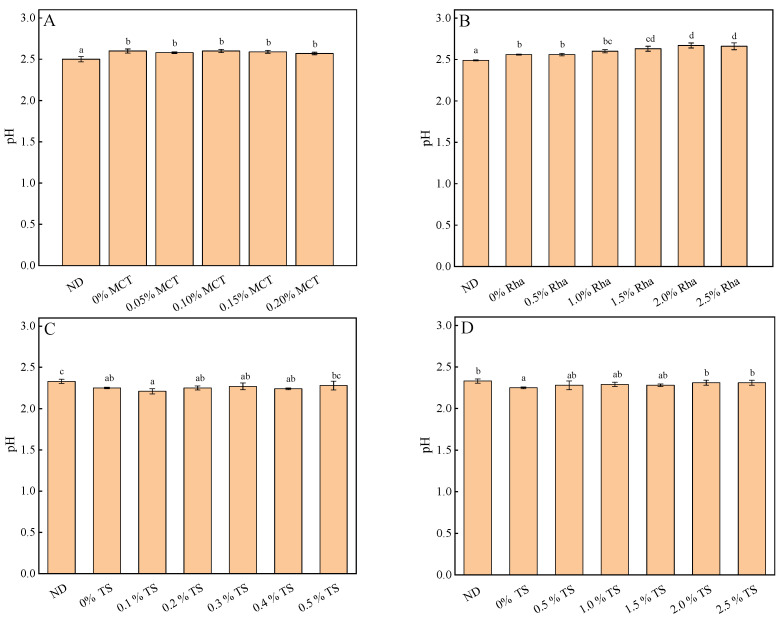
The influence of different types of emulsifiers on the pH of sea buckthorn juice: (**A**), MCT; (**B**), Rha; (**C**,**D**), low-concentration TS and high-concentration TS, respectively. Notes: lower-case labels indicate the significance of differences between samples (*p* < 0.05).

The effects of different emulsifiers on the pH of the sea buckthorn juice are shown in Figure 5. When the amount of MCT is 0~0.20%, the pH value is unchanged compared with 0% MCT (shown in Figure 5A). For the samples added with Rha, the pH value increased slightly with the increase in Rha concentration (shown in Figure 5B). For the samples added with TS, there was little change in pH when the concentration of TS was lower than 1.5% (shown in Figure 5C,D). Compared with 0% TS, when the concentration of TS increased to 1.5~2.5%, the pH value increased significantly. The variation trend of pH was the same as that of TSS. Although the results of the one-way analysis of variance showed significant differences between some samples, the variation in pH between all the samples was less than 0.2 and had no significant effect on the pH of the whole sample. The pH value of sea buckthorn juice in this study was consistent with the results of other researchers [10].

### 3.4. Physical Stability

**Figure 6:**
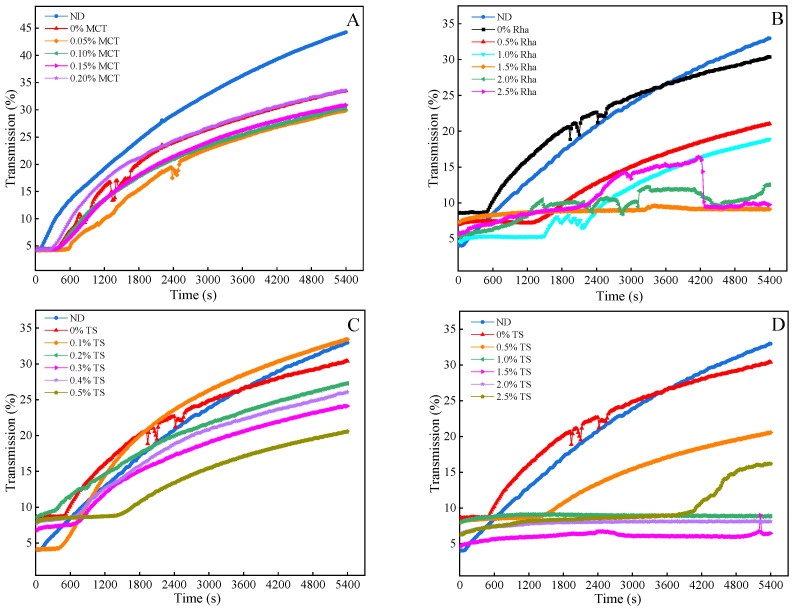
The influence of different emulsifiers on the physical stability of sea buckthorn juice: (**A**), MCT; (**B**), Rha; (**C**,**D**), low-concentration TS and high-concentration TS, respectively.

The addition amount of emulsifier is closely related to the adsorption amount, which can affect the stability of the system. The effects of different emulsifiers on the physical stability of sea buckthorn juice are shown in Figure 6. For the samples added with MCT, 0% MCT is more stable than ND. Compared with 0% MCT, the physical stability of sea buckthorn juice can be improved by adding 0.05~0.15% MCT (shown in Figure 6A). However, the physical stability decreased gradually with the increase in MCT from 0.05% to 0.20%; that is, 0.05% MCT was the most stable. For the samples added with Rha, the physical stability increased in 5400 s in the following order: ND < 0% Rha < 0.5% Rha < 1.0% Rha < 2.0% Rha < 2.5% < 1.5% Rha (shown in Figure 6B). This indicates that DHPM and the addition of Rha could both significantly improve the physical stability of sea buckthorn juice. In the range of 0% Rha~1.5% Rha, the stability improved gradually with the increase in Rha concentrations. The physical stability was reversed when the addition of Rha increased to 2.0% and 2.5%. For the samples added with TS, physical stability increased in the order of 0.1% TS ≤ ND < 0% TS < 0.2% TS < 0.4% TS < 0.3% TS < 0.5% TS < 2.5% TS < 1.0% TS < 2.0% TS < 1.5% TS (shown in Figure 6C,D). These results show that when the concentration of TS is greater than 0.2%, it can improve the physical stability of sea buckthorn juice. Additionally, when 1.0~2.0% TS is added to the juice, the slope of the transmittance curve over time is close to 0 (close to the X axis, which represents time intervals), indicating that the sample has strong physical stability. The 2.5% TS was stable in the first 60 min of centrifugation, but its physical stability decreased rapidly after 60 min (shown in Figure 6D).

Different emulsifiers and different amounts of the same emulsifier have different effects on the physical stability of sea buckthorn juice. In addition, it was found that the variation trend of physical stability is consistent with that of particle size. This is because the detection method of the stability analyzer (LUMiSizer) is based on the centrifugal acceleration principle for predicting the settling rate of suspended particles in spheres, and the settling rate of particles can reflect the stability of the system to a certain extent. Therefore, particle size is related to the physical stability of the system. For sea buckthorn juice, the addition and dissolution of Rha and TS will introduce a small number of bubbles, and the measurement process of physical stability is to collect the transmission value, so the bubbles generated in the centrifugal process will bring certain interference to the measurement of physical stability. During the emulsifying process, the emulsifier is dispersed in the form of microdroplets (micron scale) in the sea buckthorn juice system, its polar head is inserted into the water phase and the lipophilic base part is extended into the oil phase, so the interfacial tension of each component in the mixed system (sea buckthorn juice itself is a mixed unstable system) is reduced, and a solid film is formed on the surface of the microdroplets or a double electric layer is formed on the surface of the microdroplets due to the charge given by the emulsifier so as to prevent the microdroplets from converging with each other. Consequently, a uniform and stable milky liquid system is formed [22].

### 3.5. Microstructure

**Figure 7:**
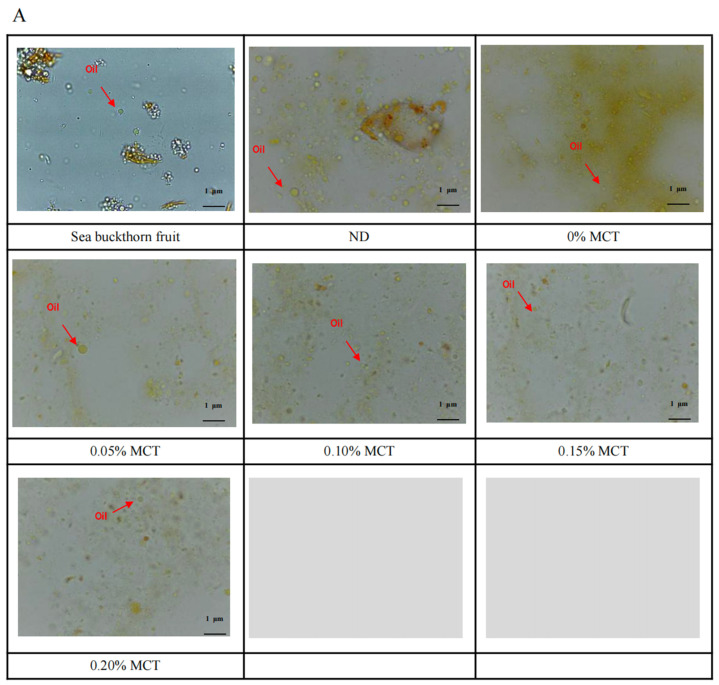

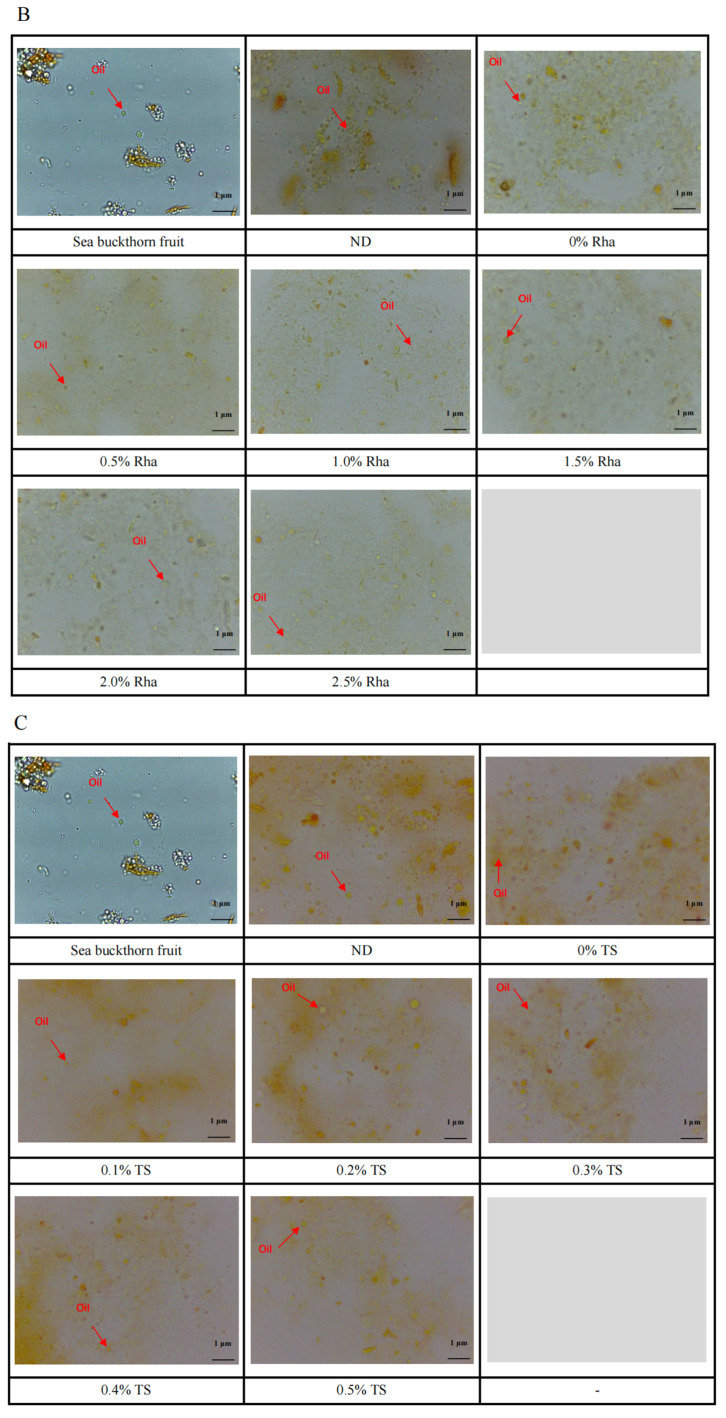

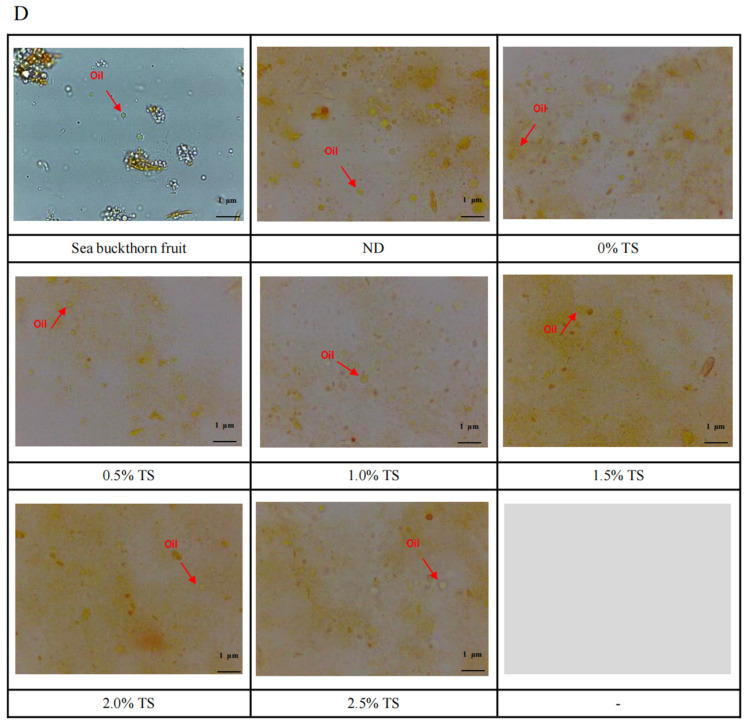
Microstructure of the sea buckthorn juice treated with DHPM and emulsifiers, objective magnification ×10: (**A**), MCT; (**B**), Rha; (**C**,**D**), low-concentration TS and high-concentration TS, respectively.

In this study, optical microscopy was used to observe the microstructure of sea buckthorn fruit and juice, sea buckthorn juice treated with DHPM, and emulsifier-contained sea buckthorn juice treated with DHPM (obtained microstructure pictures shown in Figure 7). In previous studies, lipid substances in sea buckthorn juice were stained by fluorescence staining so as to locate the storage location and state of carotenoids in the cells [44]. The results showed that the sea buckthorn tissue cells are easily damaged by the external environment because the fruit are very soft and juicy; therefore, it is difficult to observe the complete cell structure wrapped by the cell wall under a light microscope. Nevertheless, it was found that the plant cell tissues in the juice of sea buckthorn fruit after peeling were more complete compared with DHPM-treated samples, including yellow rods, green globules and fat globules, and these tissues clustered together or overlapped with each other to a certain extent (shown in Figure 7). The cell tissue in sea buckthorn juice (which refers to the sample of ND) was also relatively complete. However, the cell tissues of sea buckthorn juice treated with DHPM and combined with emulsifiers were severely damaged and reduced to fine fragments. In particular, the yellow rods were basically broken, the green balls were partially broken and some fat balls were released in the sea buckthorn juice. From the microstructure pictures, it is observed that the carotenoids in sea buckthorn juice exist in the form of free spheres surrounded by lipid droplets, and some of them are trapped by other cell tissues. Previous research observed that the size of oil droplets in the upper layer of sea buckthorn subspecies ssp [45]. Sinensis is smaller than that in ssp. mongolica cv. Indian Summer after the sample was centrifuged, and most of the oils are attached to the aggregated particles in the form of droplets. Although the raw material used in this study is the oil-removed sea buckthorn juice, the simple centrifugal de-oiling method cannot completely remove the oil in the sea buckthorn juice, and a small part of the oil still exists in the sea buckthorn juice. The content of free fat and the total content of free and bound fat were 0.27 ± 0.01 g/100 g and 0.40 ± 0.01 g/100 g, respectively, by the Soxhlet extraction method and acid hydrolysis method. Although the oil in sea buckthorn juice brings some difficulties to industrial processing, it is rich in unsaturated fatty acids, and bioactive substances such as carotenoids dissolve in oil, which plays an important role in the nutritional value of sea buckthorn juice.

According to the microstructure analysis, the microparticles in sea buckthorn juice were mostly composed of green spheres or tissue aggregates containing green spheres, which is consistent with the results of previous research [45,46].

### 3.6. Color Analysis

**Figure 8:**
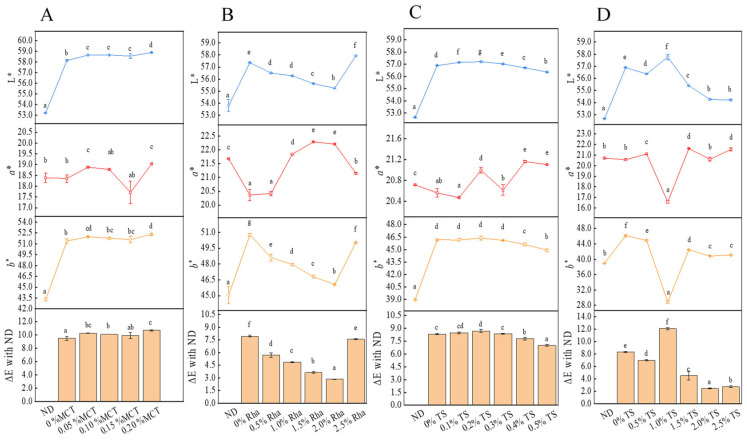
Effect of DHPM combined with emulsifier on color of sea buckthorn juice. Notes: lower-case labels indicate the significance of differences between samples (*p* < 0.05). (**A**), MCT; (**B**), Rha; (**C**,**D**), low-concentration TS and high-concentration TS, respectively.

Color is a critical quality attribute of sea buckthorn juice, primarily influenced by carotenoids. Dynamic high-pressure microfluidization (DHPM) without emulsifier addition enhances the L* (lightness) and b* (yellowness) values while reducing a* (redness) values, resulting in a brighter and less red juice (Figure 8). The addition of medium-chain triglycerides (MCTs) at concentrations of 0–0.20% further increases L* values, enhancing brightness and making the color perceptibly redder at 0.05% and 0.20% MCT compared to 0% MCT. Significant color changes (ΔE > 3.0) are observed with DHPM and MCT, indicating visible differences to the human eye [10]. In contrast, increasing concentrations of rhamnolipids (Rha) from 0.5% to 2.0% darken the juice and enhance redness, while concentrations above 2.5% brighten and increase yellowness, indicating a complex interaction between Rha concentration and color parameters. For low concentrations of tocopherol succinate (TS, 0.1–0.5%), ΔE values remain below 3.0, suggesting minimal color impact. However, higher concentrations (0.5–2.5%) result in noticeable color changes, particularly at 1.0% TS, where there is a significant decrease in lightness and yellowness and an increase in brightness.

Comparative studies show that the L*, b* and a* values of our sea buckthorn juice samples vary significantly from previous studies, likely due to differences in carotenoid content influenced by geographical and environmental factors. According to previous research, the L*, b* and a* values of sea buckthorn juice (Leikora varieties) were 47.64~47.93, 39.72~45.21 and 14.57~17.19, respectively [10]. The L* value measured in this study was far greater than the L* value measured in the previous research [47]. The a* value was greater than the a* value measured in another research [48]. The b* and L* values were closer to the results measured in the previous study [10]. The differences in the above results may be related to the differences in the chemical composition of fruit materials used for research, such as carotenoids, caused by different geographical locations and climatic environments. According to research reports, the content and structure of carotenoids can affect the color of some fruit juices [30].

### 3.7. Carotenoids Content

After adding different emulsifiers in sea buckthorn juice and treating it with DHPM, the total carotenoids content (TCC) is shown in Table 1. For the sea buckthorn juice added with MCT, the TCC was slightly decreased compared with ND and 0% MCT, but the difference was not significant. For the samples added with Rha, the TCC of 1.5% Rha increased from 10.83 ± 0.93 mg/100 g·fw to 15.73 ± 1.95 mg/100 g·fw. The trend of TCC is consistent with that of physical stability, and the reason may be related to the reduction in the particle size and the maintenance of the physical stability of the system by the emulsifier. The addition of the emulsifier changes the interface structure of the system, and emulsifiers may play a protective role in carotenoids. On the other hand, emulsifiers indirectly promote the release of carotenoids from tissue cells by reducing the size of the system [49,50]. For the sea buckthorn juice added with TS, when the concentration of TS was 0~2.5%, the TCC of 1.5% TS was significantly increased (increased from 10.83 ± 0.93 mg/100 g·fw to 14.94 ± 1.86 mg/100 g·fw) compared with ND, and the other concentrations had little effect on the TCC of sea buckthorn juice. It was found that there was a positive correlation between the concentration of Rha and TCC, which was consistent with the linear function y = 1.8189x + 11.45, R2 = 0.772. The different types and properties of emulsifiers used in this study may cause different effects on the TCC of sea buckthorn juice.

### 3.8. Bioaccessibility of Carotenoids

**Figure 9:**
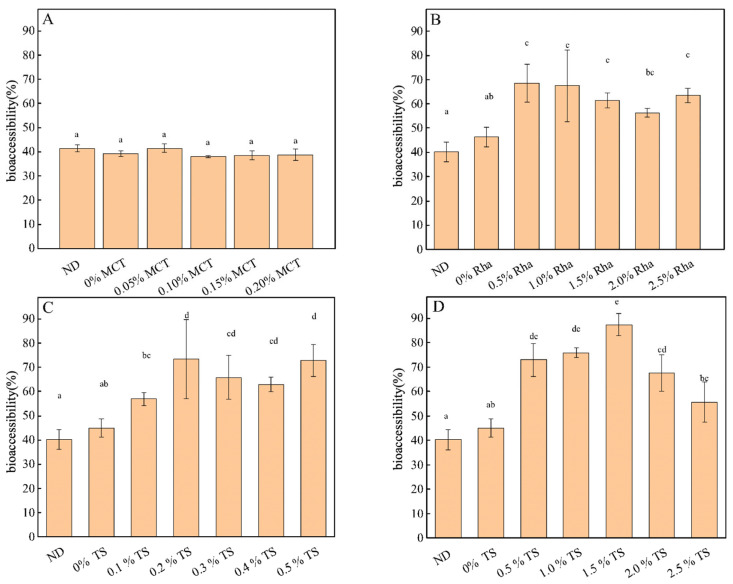
Effects of emulsifiers on bioaccessibility of carotenoids in sea buckthorn juice. Notes: lower-case labels indicate the significance of differences between samples (*p* < 0.05). (**A**), MCT; (**B**), Rha; (**C**,**D**), low-concentration TS and high-concentration TS, respectively.

The effects of different emulsifier additives on the total carotenoid bioavailability (TCB) in sea buckthorn juice are shown in Figure 9. Initially, DHPM treatment increased TCB in sea buckthorn juice compared to ND, but the difference was not significant. When 0% to 0.20% MCT was added to sea buckthorn juice, the addition of MCT did not significantly change the TCB in sea buckthorn juice (Figure 9A). The TCB in sea buckthorn juice increased significantly when 0% to 2.5% Rha was added to the juice, with 0.5% Rha having the highest TCB and a value of 68.52 ± 7.81% (Figure 9B).When 0~0.5% TS was added to sea buckthorn juice, the TCB increased from 40.24 ± 4.01% to the highest value of 73.45 ± 16.40%, in which the highest TCB was found in 0.2% TS, but the difference in TCB in 0.2~0.5% TS was not significant (Figure 9C). When higher concentrations of TS were added to sea buckthorn juice, TCB increased significantly with an increasing TS concentration in the range of 0.5% TS~1.5% TS; when the concentration of TS was increased from 1.5% to 2.0% and 2.5%, TCB decreased with an increasing TS concentration (Figure 9D). This could be related to the efficiency of nutrient transfer from the diet to the digestive fraction in relation to the structure of the dietary matrix.

It had been demonstrated that the high-pressure homogenization (HPH) treatment of citrus juice at 150 MPa increased the bioaccessibility of total carotenoids by reducing the particle size [51]. It has also been reported that polygalacturonase (PG) treatment increased the bioaccessibility of total carotenoids in carrot juice [52]. The reduction in particle size is one way to enhance the micellization of carotenoids. Additionally, the breakdown of endogenous pectin in the system may also improve the micellization of carotenoids [52]. In this study, both dynamic high-pressure microfluidization (DHPM) treatment and the addition of emulsifiers decreased the particle size, leading to a larger surface area for digestive enzymes to interact with lipid droplets during digestion, thereby enhancing the bioaccessibility of carotenoids. As emulsifiers are amphiphilic molecules, they can interact with various polar and nonpolar components commonly present in foods, such as water, carbohydrates, proteins, fats, oils and flavors [53]. Small-molecule emulsifiers can influence the oil/water interface by partially or completely replacing proteins [54]. Therefore, the Rha and TS used in this study enhance the bioaccessibility of carotenoids by modifying the interface of the sea buckthorn juice system.

## 4. Conclusions

The addition of MCT, Rha and TS in sea buckthorn juice could significantly reduce particle size and size distribution, improve TSS, turbidity, physical stability and carotenoid content and improve color parameters. It has also been proven that the combination of DHPM and emulsifier has a synergistic effect on particle size and carotenoid content. However, the opposite effect can also occur for turbidity. The release rate of carotenoids was increased by combining the DHPM treatment with emulsifiers, but the DHPM treatment decreased the turbidity of the sea buckthorn juice while the emulsifiers had the opposite effect (increased turbidity). The DHPM treatment preserved the bioavailability of all the carotenoids in the sea buckthorn juice. Among the three different emulsifiers (MCT, Rha and TS) selected in the study, Rha and higher concentrations of TS were able to improve the bioaccessibility of total carotenoids in sea buckthorn juice. The changes in physical and chemical indices of sea buckthorn juice were related to DHPM treatment and the emulsifier type and amount added. In summary, among the three emulsifiers (MCT, Rha and TS), Rha and TS were more effective than MCT in improving the quality and nutritional value of sea buckthorn juice. The combination of DHPM treatment with emulsifiers improved the quality of the sea buckthorn juice and increased the carotenoid content and bioaccessibility.

## Figures and Tables

**Table 1:** Total carotenoids content (TCC) of sea buckthorn juice added with different emulsifiers.

	Total Carotenoids Content mg/100 g fw		Total Carotenoids Content mg/100 g fw
MCT		Low-concentration tea saponion	
ND	9.1057 ± 1.1717 ^a^	ND	10.8329 ± 0.9316 ^ab^
0% MCT	8.3488 ± 0.5982 ^a^	0% TS	11.4504 ± 1.1880 ^b^
0.05% MCT	7.8128 ± 0.2189 ^a^	0.1% TS	8.0607 ± 0.5908 ^a^
0.10% MCT	7.8057 ± 0.2256 ^a^	0.2% TS	10.5680 ± 2.3076 ^ab^
0.15% MCT	7.8106 ± 0.2145 ^a^	0.3% TS	9.9941 ± 1.4994 ^ab^
0.20% MCT	7.8081 ± 0.2246 ^a^	0.4% TS	8.9610 ± 0.8435 ^ab^
		0.5% TS	10.7030 ± 2.7887 ^ab^
Rha		High-concentration tea saponion	
ND	10.8329 ± 0.9316 ^a^	ND	10.8329 ± 0.9316 ^a^
0% Rha	11.4504 ± 1.1880 ^a^	0% TS	11.4504 ± 1.1880 ^a^
0.5% Rha	11.5136 ± 0.9405 ^a^	0.5% TS	10.7030 ± 2.7887 ^a^
1.0% Rha	12.9001 ± 1.0776 ^ab^	1.0% TS	11.4544 ± 0.3444 ^a^
1.5% Rha	15.7299 ± 1.9474 ^b^	1.5% TS	14.9443 ± 1.8581 ^b^
2.0% Rha	15.5801 ± 2.9917 ^b^	2.0% TS	11.6394 ± 1.4806 ^a^
2.5% Rha	14.9905 ± 0.8207 ^b^	2.5% TS	12.3089 ± 0.6041 ^ab^

Notes: the values are the mean ± standard variance (*n* = 3). Small letters represent significant differences within the same column (*p* < 0.05). “fw” indicates “fresh weight”.

## Data Availability

The original contributions presented in the study are included in the article and Supplementary Materials, further inquiries can be directed to the corresponding author.

## Supplementary Materials

The following supporting information can be downloaded at: https://www.mdpi.com/article/10.3390/foods13131972/s1, Table S1: The particle size of sea buckthorn juice added with emulsifiers; Table S2: Lab value for transmittance of the sea buckthorn juice added with emulsifiers.
