# Supplementary material for: Effects of Emulsifiers on Physicochemical Properties and Carotenoids Bioaccessibility of Sea Buckthorn Juice
Source: Foods. 2024 Jun 22;13(13):1972. doi: 10.3390/foods13131972 (PMC11241759; doi:10.3390/foods13131972)
Supplement: Supplementary file 1 [file foods-13-01972-s001.zip › foods-3044677-supplementary.pdf]

Table S1 The particle size of sea buckthorn juice added with emulsifiers

	$D_{[4,3]}$ μm	$D_{[3,2]}$ μm	Span
ND	8.52±0.16^{e}	1.73±0.01^{d}	11.70±0.42^{e}
0% MCT	3.61±0.36^{bc}	1.32±0.01^{a}	3.87±0.22^{bc}
0.05% MCT	4.20±0.05^{d}	1.38±0.01^{c}	5.49±0.06^{d}
0.10% MCT	3.72±0.16^{c}	1.34±0.01^{b}	4.08±0.17^{bc}
0.15% MCT	3.23±0.28^{b}	1.35±0.01^{b}	4.80±0.34^{c}
0.20% MCT	2.70±0.16^{a}	1.33±0.01^{a}	3.07±0.12^{a}
0% Rha	2.80±0.09^{c}	1.35±0.01^{e}	3.26±0.20^{c}
0.5% Rha	1.90±0.07^{b}	1.18±0.01^{d}	2.40±0.13^{b}
1.0% Rha	1.51±0.05^{a}	1.02±0.01^{a}	1.79±0.10^{a}
1.5% Rha	1.77±0.07^{b}	1.07±0.02^{b}	2.14±0.19^{b}
2.0% Rha	1.85±0.05^{b}	1.11±0.02^{c}	2.13±0.12^{b}
2.5% Rha	1.92±0.02^{b}	1.13±0.01^{c}	2.27±0.05^{b}
0% TS	3.56±0.10^{d}	1.47±0.006^{d}	3.65±0.12^{d}
0.1% TS	3.32±0.05^{c}	1.33±0.005^{c}	4.31±0.15^{c}
0.2% TS	2.65±0.05^{a}	1.27±0.004^{b}	3.33±0.11^{a}
0.3% TS	2.63±0.04^{a}	1.26±0.003^{ab}	3.25±0.07^{a}
0.4% TS	2.72±0.02^{ab}	1.26±0.003^{a}	3.41±0.03^{ab}
0.5% TS	2.87±0.20^{b}	1.33±0.012^{c}	3.47±0.23^{b}
1.0% TS	2.63±0.02^{c}	1.23±0.007^{c}	3.14±0.05^{c}
1.5% TS	2.10±0.02^{b}	1.15±0.008^{b}	2.48±0.06^{b}
2.0% TS	1.86±0.05^{a}	1.09±0.012^{a}	2.34±0.08^{a}
2.5% TS	1.89±0.06^{a}	1.10±0.014^{a}	2.28±0.10^{a}

Notes: the values are the mean ± standard variance (n=3). Small letters represent the significance (p<0.05) of the difference within the same column of the same emusiflier.

Table S2 Lab value for transmittance of the sea buckthorn juice added with emulsifiers

emulsifier		a	b	L	ΔE
	ND	18.394±0.220^{b}	43.293±0.214^{a}	53.216±0.023^{a}	-
MCT	0% MCT	18.357±0.176^{b}	51.388±0.322^{b}	58.139±0.046^{b}	9.476±0.298^{a}
	0.05% MCT	18.879±0.014^{c}	51.976±0.021^{cd}	58.647±0.014^{c}	10.253±0.022^{bc}
	0.10% MCT	18.777±0.025^{ab}	51.774±0.116^{bc}	58.653±0.014^{c}	10.082±0.010^{b}
	0.15% MCT	17.708±0.530^{ab}	51.581±0.465^{bc}	58.548±0.240^{c}	9.889±0.481^{ab}
	0.20% MCT	19.034±0.001^{c}	52.305±0.106^{d}	58.887±0.018^{d}	10.667±0.084^{c}
	0% Rha	20.336±0.201^{a}	50.772±0.127^{g}	57.352±0.026^{e}	7.933±0.107^{f}

	0.50% Rha	20.422±0.077^{a}	48.605±0.319^{e}	5.502±0.048^{d}	5.691±0.243^{d}
Rha	1.00% Rha	21.841±0.032^{d}	47.94±0.097^{d}	56.261±0.027^{d}	4.875±0.06^{c}
	1.50% Rha	22.286±0.012^{e}	46.795±0.125^{c}	55.607±0.022^{c}	3.624±0.107^{b}
	2.00% Rha	22.206±0.016^{e}	46.046±0.010^{b}	55.246±0.003^{b}	2.831±0.009^{a}
	0% TS	20.561±0.079^{ab}	46.162±0.073^{d}	56.892±0.018^{d}	8.329±0.067^{c}
	0.10% TS	20.472±0.011^{a}	46.155±0.152^{d}	57.153±0.011^{f}	8.461±0.122^{cd}
	0.20% TS	20.995±0.052^{d}	46.373±0.244^{d}	57.211±0.033^{g}	8.679±0.188^{d}
	0.30% TS	20.615±0.101^{b}	46.111±0.036^{d}	57.027±0.032^{e}	8.354±0.046^{c}
	0.40% TS	21.160±0.018^{e}	45.613±0.1854^{c}	56.705±0.016^{c}	7.774±0.151^{b}
TS	0.50% TS	21.104±0.0081^{e}	44.917±0.156^{b}	56.370±0.010^{b}	7.004±0.127^{a}
	1.00% TS	16.565±0.1404^{a}	28.835±0.291^{a}	57.760±0.215^{f}	12.094±0.164^{f}
	1.50% TS	21.616±0.016^{d}	42.479±0.085^{d}	55.396±0.002^{c}	4.524±0.695^{c}
	2.00% TS	20.610±0.179^{b}	40.852±0.089^{c}	54.272±0.059^{b}	2.468±0.096^{a}
	2.50% TS	21.524±0.154^{d}	41.102±0.197^{c}	54.212±0.045^{b}	2.745±0.125^{b}

Note: Values are mean±standard variance (n=3); small letters indicate significance of differences within the same column ($p<0.05$).
